# Combinatorial Low Dose Arsenic Trioxide and Cisplatin Exacerbates Autophagy via AMPK/STAT3 Signaling on Targeting Head and Neck Cancer Initiating Cells

**DOI:** 10.3389/fonc.2020.00463

**Published:** 2020-04-15

**Authors:** Wei-Chun Hu, Wan-Huai Teo, Tung-Fu Huang, Te-Chang Lee, Jeng-Fan Lo

**Affiliations:** ^1^Institute of Oral Biology, National Yang-Ming University, Taipei, Taiwan; ^2^School of Medicine, National Yang-Ming University, Taipei, Taiwan; ^3^Department of Orthopedics and Traumatology, Taipei Veterans General Hospital, Taipei, Taiwan; ^4^Department of Exercise and Health Science, National Taipei University of Nursing and Health Sciences, Taipei, Taiwan; ^5^Institute of Biomedical Sciences, Academia Sinica, Taipei, Taiwan; ^6^Department of Dentistry, School of Dentistry, National Yang-Ming University, Taipei, Taiwan; ^7^Cancer Progression Research Center, National Yang-Ming University, Taipei, Taiwan; ^8^Department of Dentistry, Taipei Veterans General Hospital, Taipei, Taiwan

**Keywords:** head and neck cancer-initiating cells, arsenic trioxide, combination index, autophagy, synergistic effects

## Abstract

Head and neck squamous cell carcinoma (HNSCC) is a highly lethal disease with high-level of epidemic both in the world and Taiwan. Previous studies support that head and neck cancer-initiating cells (HN-CICs), a subpopulation of cancer cells with enhanced stemness properties, contribute to therapy resistance and tumor recurrence. Arsenic trioxide (As_2_O_3_; ATO) has shown to be an effective anti-cancer drug targeting acute promyelocytic leukemia (APL). Combinatorial treatment with high dose of ATO and cisplatin (CDDP) exert synergistic apoptotic effects in cancer cell lines of various solid tumors, however, it may cause of significant side effect to the patients. Nevertheless, none has reported the anti-cancerous effect of ATO/CDDP targeting HN-CICs. In this study, we aim to evaluate the low dose combination of ATO with conventional chemo-drugs CDDP treatment on targeting HN-CICs. We first analyzed the inhibitory tumorigenicity of co-treatment with ATO and chemo-drugs on HN-CICs which are enriched from HNSCC cells. We observed that ATO/CDDP therapeutic regimen successfully synergized the cell death on HN-CICs with a Combination Index (CI) <1 by Chou-Talalay's analysis *in vitro*. Interestingly, the ATO/CDDP regimen also induced exaggerated autophagy on HN-CICs. Additionally, this drug combination strategy also empowered both preventive and therapeutic effect by *in vivo* xenograft assays. Finally, we provide the underlying molecular mechanisms of ATO-based therapeutic regimen on HN-CICs. Together, low dose of combinatorial ATO/CDDP regimen induced cell death as well as exacerbated autophagy via AMPK-STAT3 mediated pathway in HN-CICs.

## Background

Head and neck cancer (HNC), a disease with a major worldwide burden. About 95% of HNC is squamous cell carcinoma (HNSCC) which make up the sixth most common cause of cancer death. It is responsible for 300,000 deaths annually (1). HNSCC is considered one of the most common cancers leading to significant mortality and morbidity in Taiwan. Treatment for HNSCC usually involves therapies with surgery, radiation, or chemotherapy alone or concurrent chemotherapy/radiation. However, the overwhelming majority of HNSCC patients' survival outcomes still remain poor. The 5-year overall survival rate of HNSCC's patients is about 40–50% (2), thus, illustrating the urgent need to develop novel therapeutic options to prolong the patients survival.

In the past decade, the hierarchical model of cancer stem cells (CSCs) or cancer initiating cells (CICs) has raised an intense topic in cancer biology or treatment. The CICs are a subpopulation of cancer cells with differentiation ability to maintain the bulk cells population of the tumor with heterogeneous phenotypes and to trigger tumor-initiating activity. Besides, the self-renewal ability of CICs also attributes to the cancer relapse, drug resistance and radio-resistance (3). Most of the therapeutic agents are capable of eliminating the more rapidly proliferating bulk cells, however, the CICs may lay dormant after the therapies (4). After the interruption of treatment, CICs may remerge as they are intrinsically resistant to the therapy agents or they have required mutations that confer resistance to the therapy agents (5). Previously, we have successfully enriched HN-CICs by spheroid cultivation (6), and have identified a subset of HN-CICs with low intracellular reactive oxygen species levels (ROS^Low^) which sustain the stemness properties and tumorigenicity (7). The HN-CICs have demonstrated chemo-resistant phenotype. Interestingly, this subpopulation cells also can be enriched in cisplatin-resistance cell line (7). Thus, development of novel therapeutic agents to target HN-CICs is required, and it would benefit for future HNSCC therapy.

Arsenic Trioxide As_2_O_3_ (ATO) has been used in traditional Chinese medicine as pharmaceutical agents for over 2400 years (8). It has shown promising results in the treatment of hematopoietic malignancies (9, 10). In year 2000, Food and Drug Administration (11) have approved arsenic trioxide (Trisenox^TM^) for treatment of acute promyelocytic leukemia (APL) (12). Studies have shown that ATO was an effective therapeutic agent in APL with 63.8 to 93% high remission rate and could prolong patients' survival rate (13). A number of studies also have revealed a pro-apoptotic activity of ATO in solid tumors, including breast cancer, gastric cancer, hepatocellular carcinomas and sarcoma (14–17). Although ATO is able to improve the disease outcome, of note, ATO also introduces to common side effects such as gastrointestinal disorders, cough, fatigue, skin rash, myelosuppression and the most burden are the liver function failure and cardiac toxicity (18). Previous studies have revealed that ATO has been correlated to many anticancer mechanisms, such as promoting tumor cell differentiation, inhibition of tumor cell growth and induce cells apoptosis. ATO also shows to reduce chemo-resistance in tumor cells via inducing the apoptosis mechanism (19). ATO has been reported to induce partial differentiation and apoptosis in APL cells (20, 21). Although ATO anti-cancer activity has utterly studied in various solid tumors such as hepatocellular carcinoma (HCC), colorectal cancer (CRC), breast cancer and gliomas, it's only effective in APL treatment and less successful in other malignancies. Clinically, high dose of ATO is required to accomplish the anti-cancer activity in solid tumors in comparison with hematological malignancies (22). Instead of attempting on ATO single treatment, researchers are looking toward the combinatorial therapeutic regimen. It has been reported that ATO enhances the therapeutic efficacy of cisplatin treatment on both oral and ovarian cancers (23, 24). ATO treatment has been carried out in HNSCC (25, 26), however the results indicating that high doses of single ATO is required to eliminate the cancer cells. Impractical high doses usage of ATO will cause strong side effects in heart and vascular toxicity. Concerning these side effects, the combination regimens of ATO treatments are applied to various cancers (27).

ATO can induce autophagy cell death in various solid tumors (28). Autophagy is referred to an intracellular degradation system in cytoplasmic components and act as cytoprotective to overcome various stress condition. The interplay of autophagy and cancer is complicate and remain controversy. There are evidences implicating that autophagy may play a role as a tumor suppressor (29), while some suggest that it may promote tumorigenesis (30). Interestingly, our previous finding shows that YMGK-1 from *Antrodia cinnamomea* successfully eliminates HN-CICs via autophagy mediated cell death (31).

In this study, we performed a combinatorial low dose ATO/cisplatin (CDDP) treatment targeting the HN-CICs as well as HNSCC cisplatin-resistant cells (HNSCC-CisPt^R^). We examined the cytotoxicity effects of low dose ATO/CDDP treatment both *in vitro* and *in vivo* assays. The experimental results revealed that the combinatorial of low dose ATO/CDDP treatment has a great potential to promote cell death in HN-CICs. In addition, we further investigated the cellular mechanism underlying ATO-base therapeutic regimen induced cell death. We found that ATO/CDDP not only induced cell differentiation but also exaggerated autophagy mediated cell death. The combinatorial low dose of ATO/CDDP treatment provided a potential therapeutic application, which can efficiently eradicate the HN-CICs.

## Materials and Methods

### Cell Lines Cultivation and Enrichment of HN-CICs From HNSCCs

The oral cavity HNSCC cell lines, SAS obtained from Japanese Collection Research Bioresources (Tokyo, Japan), OECM1 provided by Prof. Ching-Liang Meng of National Defense Medical College, (Taipei, Taiwan) and SAS-CisPt^R^ cells were used in this study. SAS, SAS-CisPt^R^ and OECM1 cells were cultured in DMEM and RPMI supplemented with 10% FBS (GIBCO, Mexico), respectively (6, 7). The enrichment of HN-CICs were performed by cultivating both cell lines in tumor sphere condition medium consisting of serum-free DMEM/F12 medium (GIBCO, UK), N2 supplement (GIBCO, USA), 10 ng/mL human recombinant basic fibroblast growth factor (bFGF), and 10 ng/mL Epidermal Growth Factor (EGF) (PEPROTECH, USA). The cells were plated at a density of 7.5 × 10^4^ live cells per 100 mm dishes as per experimental requirement. The cells were monitored and the medium was changed every other day until the tumor sphere cells were formed in about 4 weeks. All cells were cultured under the condition of 37^o^C with 5% CO_2_ (6).

### Western Blot

Protein extracts were prepared from cells by using RIPA buffer, and the protein concentration was measured by protein assay kit (Bio-Rad, USA). Protein extracts were denatured in sample buffer and subjected to SDS-PAGE gel electrophoresis. The electrophoretic proteins were then transferred to the nitrocellulose (NC) membrane. Nitrocellulose membranes were blocked in 5% skimmed milk and probed with primary antibodies. NC membrane were then washed and incubated with HRP-conjugated secondary anti-rabbit IgG or anti-mouse IgG at room temperature in TBST containing 5% milk for 1 h. After extensive washes in TBST, the signals were visualized by the enhanced chemiluminescence system as described by the manufacturer (Millipore, Germany) in conjunction with in LAS-4000 image analyzer (GE Healthcare, Japan). The immunoblotting signals from anti-Beta-actin (BA3R, Thermo Fisher Scientific, USA) or anti-GAPDH (GA1R, Thermo Fisher Scientific, USA) antibodies were used as a loading control.

### Annexin V Apoptotic Assay

Apoptotic cells were detected with an Annexin V-FITC kit (Calbiochem, Darmstadt, Germany). 1 × 10^6^ cells were stained with Annexin V–FITC and analyzed by FACS Calibur apparatus (Becton Dickinson, USA).

### Anchorage Independent Growth Assay

Each well (35 mm) of a six-well culture dish was coated with 2 ml bottom agar (Sigma-Aldrich, USA) mixture [DMEM, 10% (v/v) FCS, 0.6% (w/v) agar]. After the bottom layer was solidified, 1 ml top agar-medium mixture [DMEM, 10% (v/v) FCS, 0.3% (w/v) agar] containing 1 × 10^4^ cells with ATO or CDDP single treatment and ATO/CDDP combined treatment was added, and the dishes were incubated at 37°C for 15 days. The colonies were counted over five fields per well for 15 fields in triplicate experiments.

### Subcutaneous Xenografts in Nude Mice

All the animal practices in this study were approved and treated in accordance with the Institutional Animal Care and Use Committee (IACUC No. 1020504) of National Yang-Ming University, Taipei, Taiwan. HN-CICs cells were subcutaneously injected into BALB/c nude mice (6–8 weeks). Tumor volume (TV) was calculated using the following formula: TV (cm^3^) = (Length × Width ^2^)/2.

### Inmmunohistochemistry

After deparaffinization and rehydration, the tissue sections were processed with antigen retrieval by boiling the slides in sodium citrate buffer (10 mM, pH 6.0). The slides were immersed in 3% H_2_O_2_ for 10 min and washed thrice with PBST. The tissue sections were then blocked with serum (Vestastain Elite ABC kit, Vector Laboratories, USA) for 30 min, followed by incubating with the primary antibody, in PBS solution at room temperature for 2 h in a container. Tissue slides were washed with PBS and incubated with biotin-labeled secondary antibody for 30 min, followed by 30 min streptavidin-horse radish peroxidase conjugates incubation. The slides were washed thrice with PBS. Subsequently, the tissue sections were immersed with AEC substrate kit as described by the manufacturer (Dako Corporation, USA) for 10 min. Hematoxylin was applied for counter-staining. Finally, the tumor sections were mounted with Gurr® (BDH Laboratory Supplies, U.K.) and examined under a microscope.

## Results

### Combinatorial Low Dose ATO/CDDP Treatment Synergistically Promotes Cell Death in HN-CICs and Diminishes the Stemness Properties

**Figure 1:**
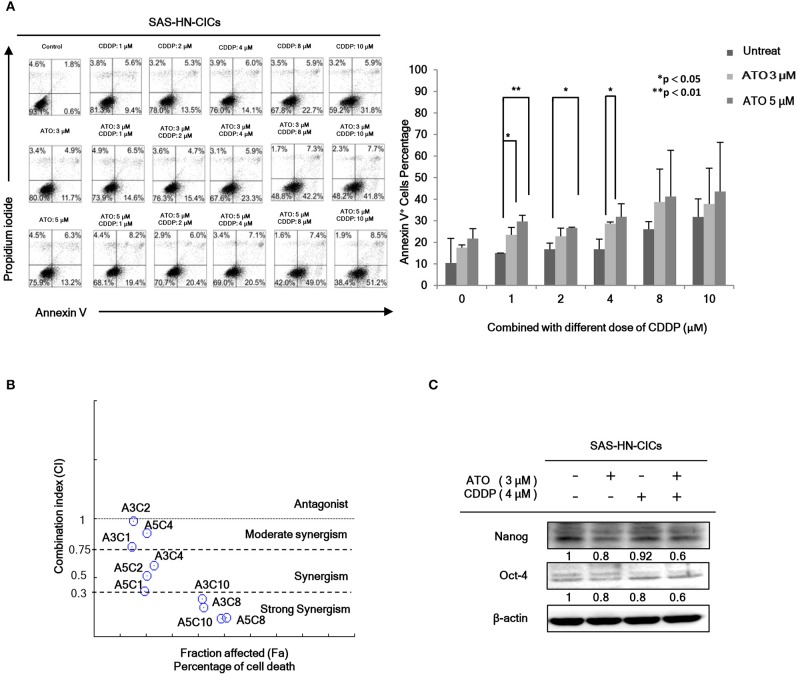
Synergistic effect of combinatorial low dose ATO/CDDP treatment on promoting cell death and diminishing the stemness properties of HN-CICs. **(A)** HN-CICs treated with ATO/CDDP, ATO, and CDDP alone for 24 h, respectively. The cell death was examined by Annexin V/PI staining. Statistical analysis was performed by student's *t*-tests. *p < 0.05 and **p < 0.01. **(B)** Synergistic effect of combination ATO/CDDP on HN-CICs was calculated by using Chou–Talalay analysis. Different doses of combination ATO/CDDP treatment induced cell death of HN-CICs (percentage) and combination index (CI value), Y axis. Each point represents different doses of combination ATO/CDDP CI value. CI values were generated by using Compusyn Software, X axis indicates the effects (Fa, fractioned effected, % inhibition), and symbols represent CI values derived from actual data points (CI = 1, activity; CI > 1, antagonism; CI < 1, synergy). **(C)** Immunoblot assay to examine the expression of the stemness markers, Nanog and Oct-4, of HN-CICs after single or combined treatment of ATO (3 μM) or CDDP (4 μM) on HN-CICs for 48 h, respectively. β-actin signal was used as loading control.

In order to evaluate the anti-cancerous efficacy of low dose combinatorial treatment with ATO/CDDP, we performed the co-treatment on HN-CICs by combining different doses of ATO with different doses of conventional chemo-drug, CDDP. Annexin V/PI double staining was used to examine the apoptotic effects by ATO/CDDP co-treatment. The flow cytometry analyses indicated that the cell number of Annexin V/PI positive staining under the co-treatment of ATO/CDDP was substantially higher than that of HN-CICs with single treatment (Figure 1A). Cells treated with low dose of ATO (3 μM) in combination with 8 μM CDDP revealed strong cell death. These results indicate that low dose of combinatorial ATO/CDDP can promote cell death of HN-CICs. However, we found that co-treatment of ATO/CDDP did not cause cell death in normal human keratinocyte cells (NHOK) (Supplementary 1). In additional, either SAS-CICs or OECM1-CICs showed more resistant to ATO single treatment when compared with parental cell lines (data not shown). To determine the synergistic effects of the combinatorial ATO/CDDP treatment, we performed the Chou-Talalay's method analyses, and the combination index value (CI value) was calculated by using CompuSyn software. As shown in Figure 1B and Table S1, most of combinational regimens were reside on synergism sections (CI < 1). For the following experiments conducted we used the combinatorial ATO/CDDP dosages with synergism. Additionally, the protein level of stemness markers such as Nanog and Oct4 was downregulated in ATO/CDDP co-treated SAS derived HN-CICs (SAS-CICs) in comparison to that of the untreated cells or cisplatin/ATO single treated cells (Figure 1C). The above mentioned findings indicate that the stemness properties of SAS-CICs were abrogated after the ATO/CDDP combinatorial treatment.

### ATO/CDDP Treatment Re-sensitizes the Cisplatin-Resistance Cell Line

**Figure 2:**
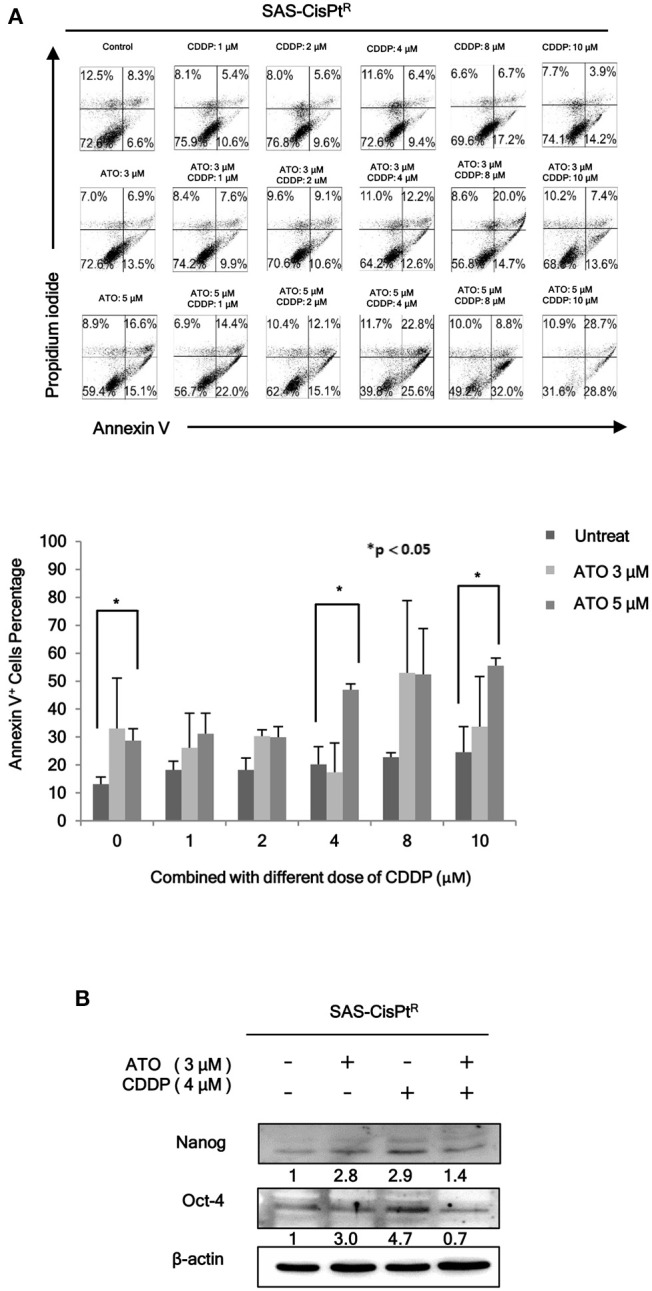
Low dose ATO/CDDP co-treatment re-sensitizing the SAS-CisPt^R^ cells. **(A)** Cell death of the SAS-CisPt^R^ cells treated with single or combined ATO and CDDP for 24 h was examined by Annexin V/PI staining. Statistical analyses were performed by student's *t*-tests. *p < 0.05. **(B)** Immunoblot analyses were used to detected the expression profile of stemness markers, Nanog and Oct-4, of SAS-CisPt^R^ cells after single or combined treatment of ATO (3 μM) or CDDP (4 μM), respectively. β-actin signal was used as loading control.

As we have demonstrated that HN-CICs are more chemoresistant (7). Here, we would like to investigate whether combinatorial low dose ATO/CDDP treatment can re-sensitize the chemo-resistant cells by co-treating the Cisplatin resistant SAS-CisPt^R^ cells (7) which possessing the HN-CICs characters with ATO/CDDP regimen. The combinatorial low dose ATO/CDDP treatment not only re-sensitized to CDDP but also induced cell death on the treated SAS-CisPt^R^ cells (Figure 2A). Consistent to SAS-CICs results, the stemness properties of SAS-CisPt^R^ cells were also diminished after the combined treatment with ATO/CDDP (Figure 2B).

### ATO/CDDP Treatment Induces Autophagic Cell Death and Promotes Cell Differentiation in HN-CICs

**Figure 3:**
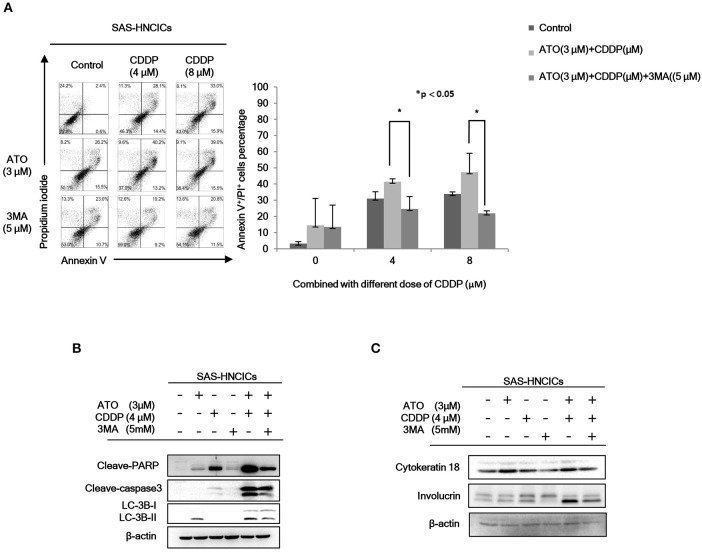
Low dose ATO/CDDP co-treatment inducing autophagy and differentiation in HN-CICs. **(A)** SAS-CICs cells were treated with different doses of ATO, CDDP and/or 3-Methylamphetamine (3-MA) for 24 h and analyzed by annexin V/PI staining. The bar chart revealed the amount of Annexin V^+^/PI^+^ cells. **(B)** Immunoblot analyses indicated expression of autophagy related markers such as PARP, cleaved caspase3 and LC3B. **(C)** SAS-CICs cells were treated with single or combined treatment of ATO (3 μM), CDDP (4 μM), and/or 3-MA (5 mM) for 48 h. Immunoblot analyses were used to detected the expression profile of epithelial differentiation marker such as Cytokeratin 18 and Involucrin. β-actin signal was used as loading control. Statistical analyses were performed by student's *t*-tests. *p < 0.05.

According to our previous research, autophagy mediated cell death can be a major molecular mechanism to induce cell death in HN-CICs (31). In fact, the role of ATO in promoting cellular autophagy are reported in other cancer cell line (27). Initially, we found that the cell death numbers were increased when HN-CICs were treated with low dose ATO/CDDP through Annexin V/PI double staining analyses. However, this induced cell death caused by ATO/CDDP co-treatment was reversed when the autophagic cell death inhibitor, 3-Methyladenine (3-MA), was simultaneously co-treated (Figure 3A). Further, we determined the protein LC-3B I/II ratio, a autophagic marker, by immunoblotting assay (Figure 3B). ATO single treatment induced expression of autophagy marker, LC-3B-II. The expression of LC-3B-II was further elevated in cells under ATO/CDDP treatment, interestingly, addition of 3-MA also reversed the expression of LC-3B-II. Moreover, the expression of both apoptotic markers [poly (ADP ribose) polymerase (PARP1) and cleaved-caspase3] was also significantly increased, and this induction of apoptotic markers can be reversed by 3-MA co-treatment. We also observed the elevated protein level of the differentiation markers (cytokeratin 18 and involucrin) in ATO/CDDP combinatorial treated cells compared to the protein level of single treated or untreated cells (Figure 3C). Taken together, these results suggest that low dose of combinatorial ATO/CDDP promotes cell death by exaggerating autophagy to promote both cell apoptosis and differentiation.

### Combinatorial Low Dose ATO/CDDP Suppresses the Malignancy of HN-CICs *in vitro* and *in vivo*

**Figure 4:**
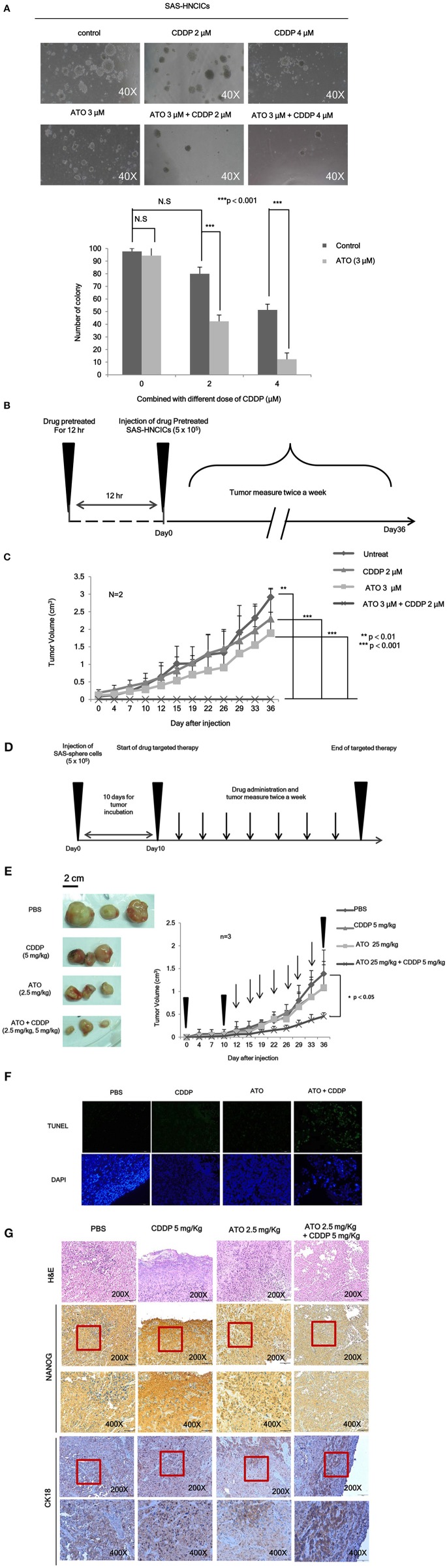
Low dose ATO/CDDP treatment suppressing the malignancy of HN-CICs both *in vitro* and *in vivo*. **(A)** SAS-CICs cells were treated with single or combined treatment of ATO and CDDP for 12 h. The treated cells were then plated on soft agar for 15 days. The colony formation ability of the SAS-CICs cell under distinct condition was collected as shown in the representative images. The bar graphs showed the amounts of colonies with a diameter ≥ 100 μm over 5 fields per assay. The data are the mean ± SD from three independent experiments and analyzed by Student's *t*-test (*p < 0.05). **(B)** The schematic of preventive model; SAS-CICs cells were singly or combinatorically pretreated with ATO (3 μM) and of CDDP (2 μM) for 12 h. These cells were subcutaneously injected into the back of nude mice. **(C)** The tumor growth generated from the treated SAS-CICs cells was recorded and analyzed. **(D)** The schematic of therapeutic model was used to demonstrate the anti-tumorous ability of low doses ATO/CDDP regimen. SAS-CICs cells were first subcutaneously inoculated onto the back of nude mice. When the tumor size reached around 0.1 cm^3^, ATO (2.5 mg/kg) and CDDP (5 mg/kg) were either singly or combinatorically intraperitoneal injected into the nude mice. PBS was used as placebo. **(E)** The mice were sacrificed on day 36, and the images of the generated tumors were collected. Tumor growth curves were also recorded and analyzed. **(F)** TUNEL assay was performed to analyze the cell death among the collected xenograft tumors. DAPI dye was used as counter staining. **(G)** Xenograft tumors generated from SAS-CICs cells treated with ATO/CDDP regimen were collected. Subsequently, immunohistochemistry staining was performed to detect the expression of stemness marker (Nanog) and differentiation marker (CK18). H&E; hematoxylin and eosin stain.

To further characterize the anti-cancerous effects of ATO/CDDP combined treatment in HN-CICs, we first performed anchorage independent growth assay. The colony number of the untreated cells was similar to that of the ATO or CDDP singly treated HN-CICs (Figure 4A). However, combined treatment with 2 or 4 μM ATO and CDDP significantly reduced the colony number (Figure 4A). These results indicate that low dose of ATO combined treatment with CDDP impairs malignancy and self-renewal ability of HN-CICs. To further elucidate the anti-cancerous growth effects of the combined treatment of ATO/CDDP *in vivo*, we used xenograft mouse models to analyze the attenuated tumorigeneity of HN-CICs by ATO/CDDP regimen treatment. Here, we carried out two animal models, the first one was administrated as the cancer preventive model (Figure 4B). To do so, the 8-week old nude mice were first subcutaneously injected with HN-CICs which were pre-treated with single or combined regimen of ATO/CDDP. The size of tumor generated from the pre-treated HN-CICs was measured continuously after cell inoculation (Figure 4C). We observed that HN-CICs under single treatment with ATO or CDDP did not significantly affect their tumor growth ability but delay the tumor initiating timing of HN-CICs during tumor formation. However, HN-CICs pretreated with ATO/CDDP regimen showed dramatic loss of tumor initiating ability. Secondly, we performed the cancer therapeutic model (Figure 4D). Nude mice were first injected with enriched HN-CICs. When mice bearing HN-CICs derived tumor, with primary tumor growing to around 0.1 cm^3^, they were administrated with single ATO, CDDP or combinatorial regimen. In the therapeutic model, we found that the effect of cisplatin single treatment was similar to the placebo group on day 36 (Figure 4E). Additionally, ATO single treatment compared to CDDP single and placebo treatment showed an anti-cancerous effect but without the statistical significance. Unsurprisingly, we observed that the combined ATO/CDDP treatment had significant effect on inhibiting tumor growth. The above mentioned results implicate that combinatorial of low dose ATO/CDDP has a therapeutic potential on targeting HN-CICs. To further verify the cell death response to drug treatment, we performed TUNEL assay to detect the dying cells of tumor mass with drug treatment (Figure 4F). The TUNEL signal was highly displayed in the combined ATO/CDDP administrated tumor sections. We also observed a strong cytokeratin 18 staining, and a mild decrease of NANOG signal on IHC staining of ATO/ CDDP combined treated tumor sections (Figure 4G). Taken together, the results suggest that low dose of ATO/CDDP therapy is able to suppress tumor growth by inducing cell death and abolishing cancer stemness in xenografted mouse model.

### The Molecular Mechanisms of ATO-Based Therapeutic Regimen Promote Cell Death in HN-CICs

**Figure 5:**
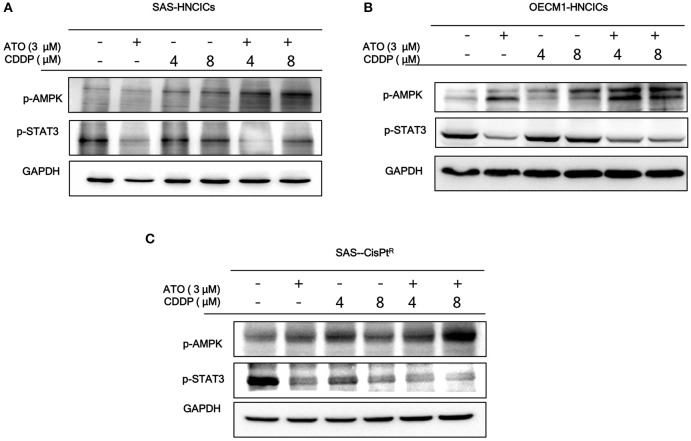
The molecular targets of ATO/CDDP regimen. **(A)** SAS-CICs, **(B)** OECM1-CICs, and **(C)** SAS-CisPtR administrated with single or combined treatment of ATO and CDDP treated were harvested and analyzed by immunoblot assay by targeting the cancerous related molecules, p-AMPK and p-STAT3. GAPDH was used as loading control.

According to our previous studies, dysregulation of autophagy could be the main molecular mechanism to induce the cell death in HN-CICs (31, 32). Further, we showed that ATO/CDDP treatment induced autophagic cell death and promotes cell differentiation in HN-CICs. Hence, we first examined the autophagy signaling pathway to uncover this molecular mechanism whether it is dysregulated in inducing cell death by ATO/CDDP regimen treatment. When the combination treatment applied, we observed a decrease of phospho-PI3K and phospho-mTOR in both SAS-HNCICs (Figure 5A) and SAS-CisPt^R^ (Figure 5C) but not in OECM1-CICs by immunoblot analyses (Figure 5B). Additionally, the ATO/CDDP combined treatment suppressed the expression of phospho-STAT3 in SAS-CICs, OECM1-CICs, and SAS-CisPt^R^. Interestingly, a significant elevation of p-AMPK was observed in these SAS-CICs, OECM1-CICs and SAS-CisPt^R^ cells under ATO/CDDP treatment. Together, the dysregulation of autophagy of HN-CICs are regulated through AMPK, STAT3 signaling pathways under ATO/CDDP treatment.

## Discussion

Arsenic trioxide is well-known of its anti-cancer activity to treat acute promyelocytic leukemia (APL) patients (33). In previous studies, 0.16 mg/kg/day ATO is administered to APL patient; the treatment course takes about 6 weeks and the dosage of ATO is nearly 6–8 μM in the plasma (34). In the conventional ATO therapy, it requires a high dose to induce the apoptotic effect. In this study, we have successfully demonstrated a low dose of combinatorial ATO/CDDP treatment synergistically induced the exaggerated autophagy mediated cell death in HN-CICs. The CI analysis indicates that the combinatorial effect of ATO/CDDP exhibits a wide range of synergism (CI < 1) in HN-CICs, ranging from 0.15 to 0.98. Further, we found that the low dose ATO (3 μM) combined with 4 μM cisplatin effectively induced apoptotic cell death in HN-CICs (Figure 1A). This combinational regimen also inhibited the HN-CICs self-renewal ability. Importantly, we observed the inhibitory tumorigenicity of HN-CICs in the therapeutic nude mice model *in vivo* which was administrated with low dose ATO/CDDP. Moreover, the combinatorial low doses of both ATO and CDDP in our study were effective even in the cisplatin-resistant cells.

HNSCC patients are still facing the relapse after therapy (35). This may due to the conventional treatments cannot efficiently eliminate CICs, which are involved in the tumor progression, metastasis, and chemo/radio resistance (36). Recent clinical studies showed that conventional chemotherapeutics generally affect proliferative cells, potentially eliminate proliferating cancer cells but not targeting the slow dividing cells CICs (37). CICs have exhibited quiescent slow-cycling phenotype and have been shown to be involved in tumor progression, cancer recurrence and metastasis because of their therapeutic resistance (31, 38). Thus, in this study we focused on ATO/CDDP combinatorial therapy targeting the HN-CICs. This ATO-based therapeutic regimen seems to promote the HN-CICs differentiation (Figures 3C, 4G) as we observed the enhanced expression of differentiation markers Cytokeratin 18 and Involucrin. It has been reported, ATO can diminish stemness properties in leukemia or hepatocellular carcinoma derived cancer initiating cells (39, 40). In addition, Tomuleasa et al. have reported that low concentration of ATO lead to differentiation in glioblastoma multiforme (GBM) stem-like cells (41). Cisplatin is a standard therapeutic agent of HNSCC through specifically targeting highly proliferation cells (2, 11, 42). Indeed, cumulative evidence suggested CDDP could not suppress the high stemness properties of cancer cells. Together, the combinatorial low dose ATO/CDDP regimen seems to facilitate HN-CICs differentiation into highly proliferated cells and subsequently underwent cell apoptosis.

We observed the induced autophagy when the ATO/CDDP was applied to HN-CICs. The expression of LC3 II (Figure 3B), a well-known marker for autophagy, was enhanced in this combinatorial treated HN-CICs. Similar results are seen in our early discovery that exaggerated induction of autophagy abolishes the stemness properties of HN-CICs, in the mean times, loss of stemness properties of HN-CICs can promote the cell differentiation ability (31). During the cancer progression and metastasis stage, autophagy emerges as a pro-tumoral role in order to eliminate the ROS-induced metabolic stress (43, 44). Intriguingly, we observed a consistency of up-regulation of p-AMPK in combinatorial treated cells. AMPK is a conserve energy-sensing kinase. AMPK is activated in response to metabolic stress and shortage of energy. Once activated, AMPK globally promotes catabolic processes. In accordance, AMPK has be linked to the regulation of autophagy (45, 46). Here, we showed that the activation of AMPK seems to increase the autophagic flux in ATO/CDDP treated cells (Figure 5). In this case, the excessive autophagy in these treated cells reverse the protective role of pro-tumoral but promoting the CICs to undergo autophagic cell death.

Another crucial issue of current HNSCC therapeutic is an emerging of drug resistance. This is most likely due to the existence of CICs which are resistant to chemo-drugs. Our previous publications (7, 32) have demonstrated that CDDP-resistance cells (SAS-CisPt^R^) consist of highly stemness properties. The combination of ATO/CDDP regimen synergistically induced cell death in SAS-CisPt^R^ cells (Figure 2A). Although the dose used to diminish the SAS-CisPt^R^ cell lines was higher than the dose used in HN-CICs, the dosage is relatively lower than clinical administrated dosage. The anti-tumorous effect of the combined ATO/CDDP treatment to ovarian cancer (24) or oral squamous cell carcinoma (OSCC) (23) has been studied by others. However, the used dose of ATO/CDDP is higher than that in our current study. Furthermore, we had successfully eradicated both the HN-CICs and SAS-CisPtR cells by using low dose of ATO/CDDP regimen.

Cisplatin induced drug resistance effects are connected with mTOR signaling up-regulated (47). Our results also revealed that the treatment of ATO/CDDP in HN-CICs could reverse the cisplatin induced mTOR upregulation in SAS-CICs and OECM1-CICs (Figure 5). Phosphatidylinositol 3-kinase (PI3K)/mammalian target of rapamycin (mTOR) pathway is a well-known signaling for controlling cell survival through autophagy regelation. This crucial survival pathway can affect cells proliferation, angiogenesis, metabolism, and differentiation. Interesting, our result revealed that the phosphorylated PI3K expression only suppressed in SAS derived HN-CICs and SAS-CisP^R^ but not in OECM1-CICs under ATO/CDDP regimen treatment. This may refer to the conversed sensitivity of PI3K between SAS-CICs and OECM1-CICs. In other autophagy inhibitor study also perform different PI3K effects during their drug treatment (48). Together, the upstream of mTOR signal may not derive from PI3K in OECM1 cells. Finally, SAS-CisPt^R^ cells did not decrease mTOR under ATO/CDDP treatment. However, PI3K signaling is becoming more sensitive in CDDP single and ATO/CDDP combined treatment in this resistance line. This phenotype may refer to drug-resistance of SAS-CisPt^R^ cells. To overcome cisplatin cytotoxicity, this cell line obtained other mTOR upstream signaling to maintain cell survive but not derived fromPI3K. However, we still can successfully promote cell death in these cells via ATO/CDDP treatment (Figure 2). The PI3K/mTOR is shown in dysregulation pathway in various cancer cells (49). Recently in glioblastoma stem-like cells research, mTOR pathway is link to the self-renewal ability and tumorigenicity of CICs. In addition, cellular key energy sensor kinase AMP activated protein (AMPK) was reported that it could promote autophagy by mTOR inactivation. In our data also suggested ATO-based therapeutic regimen can induced AMPK activity.

High concentrations of ATO can activate Jun N-terminal kinase (JNK) (50). In addition, in adipocyte study that JNK/STAT3 signaling can be suppressed by AMPK (51). In breast CICs study, STAT3 has been suggested as a specific marker, which can mediate the Nanog regulation. In our previous publication, YMGKI-2 showed to restore the chemosensitivity and promoting differentiation in CICs through targeting the Src/STAT3/c-Myc pathway (31, 32). Taken together, these evidences support that STAT3 play a critical role in HN-CICs. STAT3 signaling is constitutively activated in various cancer types (32, 52, 53). Its diverse pro-tumoral role has been widely reported. Thus, STAT3 signaling has been chosen as cancer therapeutic target. STAT3 has been proposed as CDDP sensitivity converse key factor (54). There are reports showing some potential molecules could affect the JAK-STAT3 cancerous pathway (55) to promote cell apoptosis effects. Fascinatingly, our data revealed the ATO single treatment, or ATO combined with CDDP could significantly block STAT3 activity in HN-CICs.

## Conclusion

**Figure 6:**
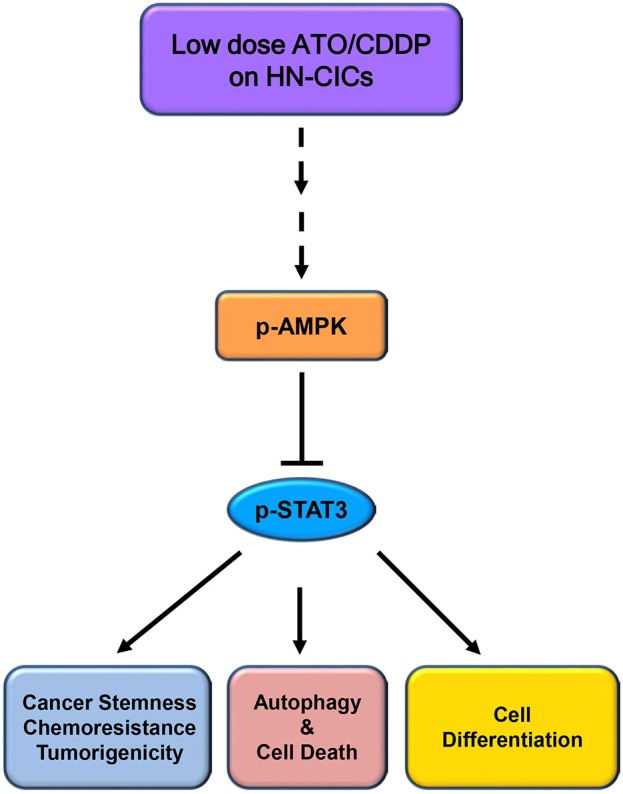
Schematic of molecular signaling targeted by low dose ATO/CDDP regimen. Overall, the low dose ATO/CDDP regimen diminishes the stemness properties, and promotes cell differentiation in HN-CICs. Exaggerated autophagy mediated cell death is induced by the low dose ATO/CDDP regimen to inhibit the tumorigenicity of HN-CICs. Tumor suppressor AMPK expression increased may participate in this molecular mechanism regulation. However, this therapeutic regimen direct or indirect targets are still unknown; (----) refers to unknown relationship or factors.

Overall, the low dose combinatorial treatment of ATO/CDDP provokes a synergistic cell death effect in both HN-CICs and SAS-CisPt^R^ cells. Although the proposed dosage is lower than the clinical dosage, it could effectively diminish the HN-CICs. Strikingly, with this low dose combinatorial ATO/CDDP, we showed either both *in vitro* and *in vivo* study, the tumorigenesis was inhibited. Finally, the ATO/CDDP treated HN-CICs and/or SAS-CisPt^R^ cells underwent cell differentiation, autophagy, and cell death might through the activation of p-AMPK and inhibition of STAT3 signaling pathway (Figure 6).

## Data Availability Statement

All datasets generated for this study are included in the article/Supplementary Material.

## Ethics Statement

The animal study was reviewed and approved by All the experimental procedures regarding animal handling were approved by the Institutional Animal Care and Use Committee (IACUC) of the National Yang-Ming University (IACUC No. 1020504).

## Author Contributions

W-CH, W-HT, and J-FL: study design and manuscript drafting. W-CH and W-HT: experimental work. T-FH, T-CL, and J-FL: reviewed the manuscript and discussed results.

### Conflict of Interest

The authors declare that the research was conducted in the absence of any commercial or financial relationships that could be construed as a potential conflict of interest.
