# Supplementary figures and images for: Combinatorial Low Dose Arsenic Trioxide and Cisplatin Exacerbates Autophagy via AMPK/STAT3 Signaling on Targeting Head and Neck Cancer Initiating Cells
Source: Front Oncol. 2020 Apr 15;10:463. doi: 10.3389/fonc.2020.00463 (PMC7174769; doi:10.3389/fonc.2020.00463)

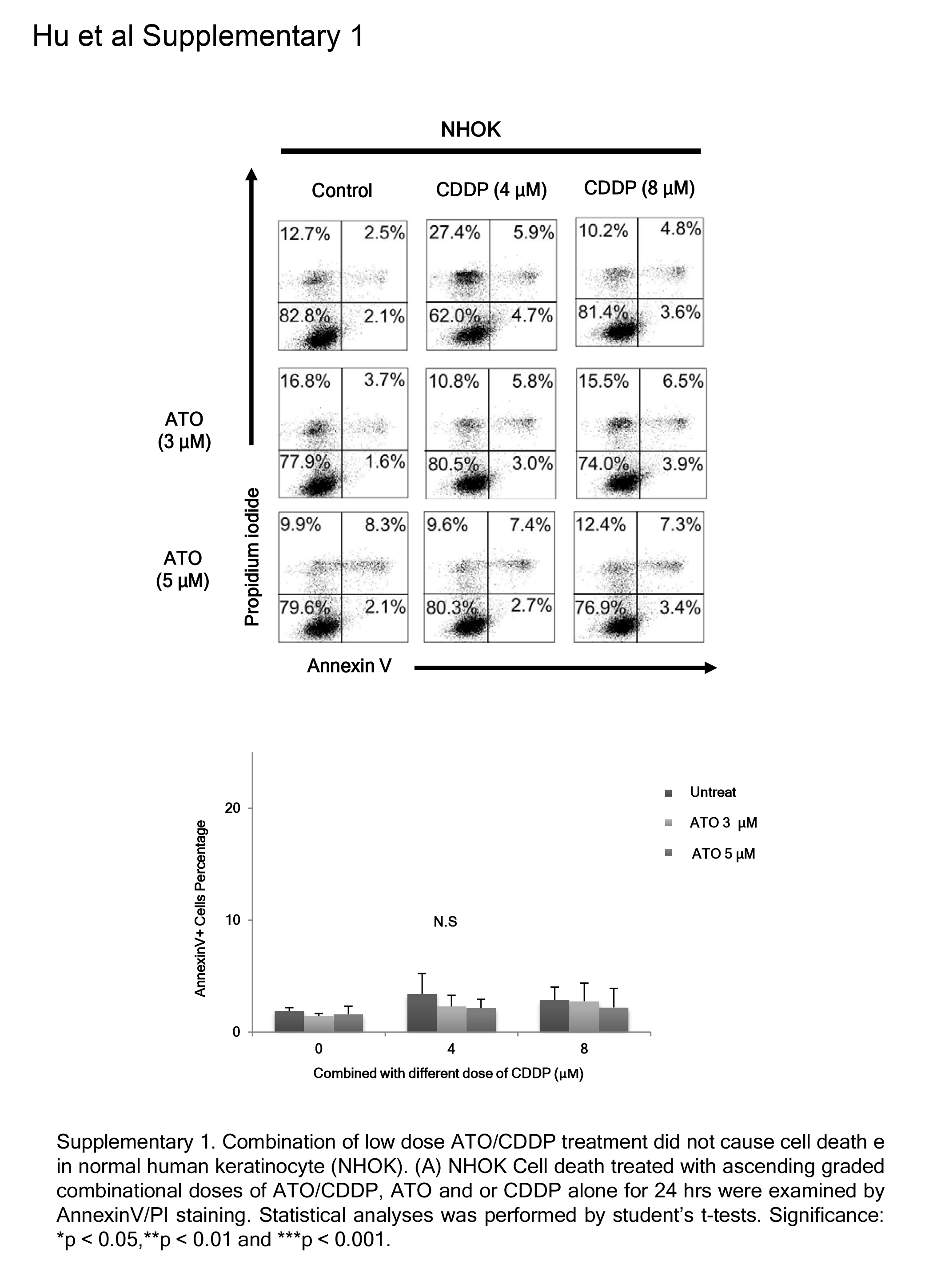

Supplement: Supplementary file 1 [file Image_1.jpg]

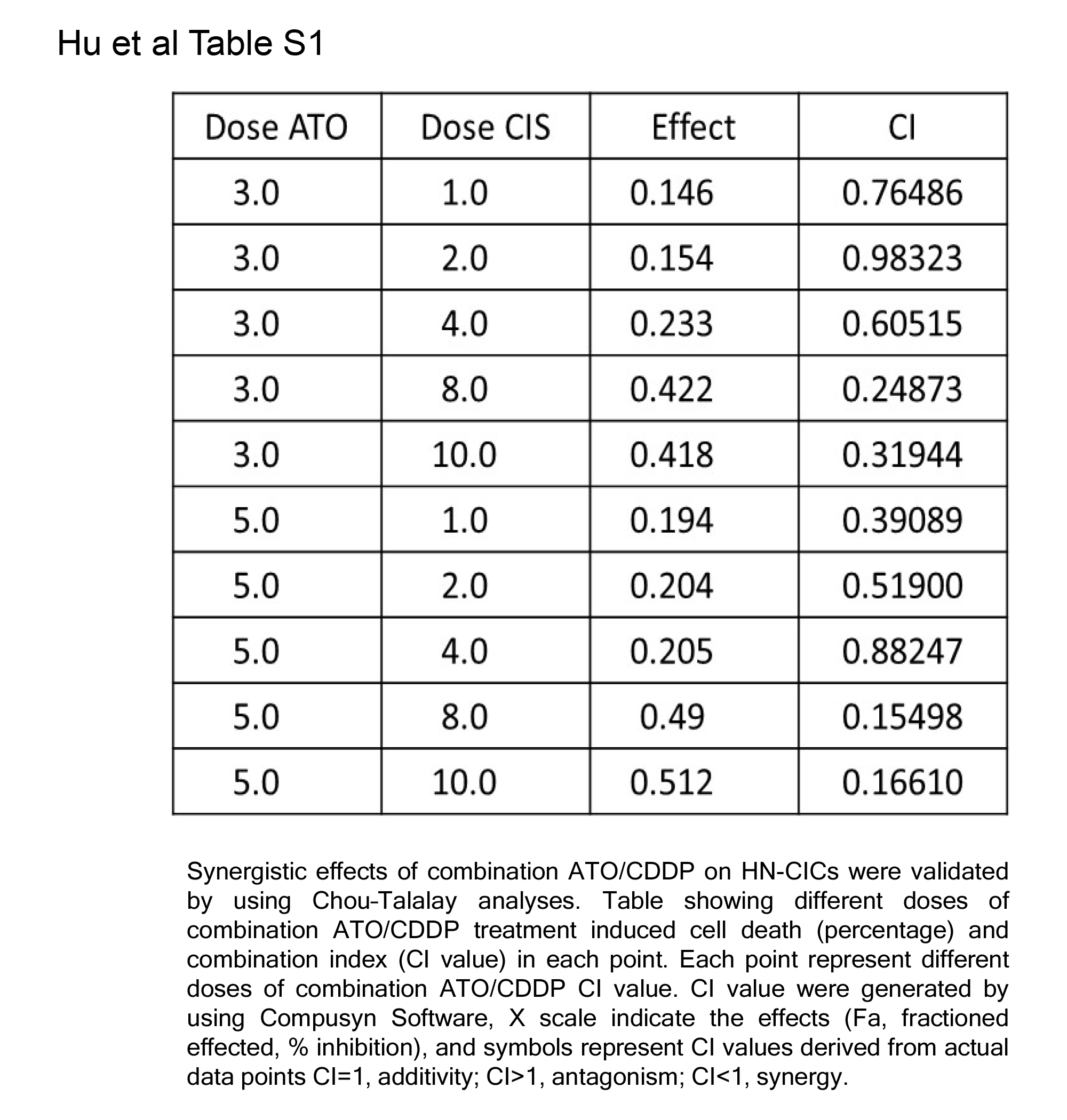

Supplement: Supplementary file 2 [file Image_2.jpg]
